# Simulation-based training for early procedural skills acquisition in new anesthesia trainees: a prospective observational study

**DOI:** 10.1186/s41077-020-00135-z

**Published:** 2020-08-12

**Authors:** Delfina Sanchez Novas, Gonzalo Domenech, Natalia Giselda Belitzky, Maria Mercedes Errecart, Sergio Adrian Terrasa, Gustavo Garcia Fornari

**Affiliations:** 1 Anesthesiology Department, Hospital Italiano de Buenos Aires, Juan D. Perón 4190, CP1199, Buenos Aires, Argentina; 2 Research Department, Hospital Italiano de Buenos Aires, Buenos Aires, Argentina

**Keywords:** Anesthesiology trainee, Competence assessment, Procedural skills, Patient safety, Simulation-based training, Assessment tools

## Abstract

**Background:**

In a setting in which learning of basic procedural skills commences upon graduation from medical school, and as a first step towards integration of simulation-based learning into the anesthesiology training program, a preparatory course for new anesthesia trainees was designed. Three educational strategies were sequentially combined (e-learning, simulation-based hands on workshops, and on-site observational learning), and performance was assessed in a stepwise approach on five procedural skills considered essential for early anesthetic management (peripheral intravenous cannulation, sterile hand wash and gowning, anesthesia workstation preparation, face-mask ventilation, and orotracheal intubation). The primary aim of this study was to determine if this preparatory training course at the onset of anesthesiology residency is useful to achieve a competent trainee performance in the clinical setting.

**Methods:**

This prospective study was carried out at a university-affiliated hospital in Buenos Aires, Argentina, from 2017 to 2019. The 24 participants, comprising three cohorts of 8 residents each, underwent a preparatory course at the onset of residency. Diverse, consecutive educational strategies, and assessments (three stages: 1, 2, 3) took place using task-specific tools (checklists) and global rating scales for five procedural skills. The primary outcome was achievement of competent scores (85%) in final assessments, and the secondary outcomes were performance improvement between assessment stages and compliance with predefined safety items.

**Results:**

Twenty trainees (83.3%) were found to be globally competent (both assessment tools for all procedures) during final assessments (stage 3). Statistically significant improvement was found for all procedural skills between baseline and after workshop assessment scores (stages 1–2), except for orotracheal intubation in checklists, and for all procedural skills between stages 2 and 3 except for sterile hand wash and gowning in checklists.

**Conclusions:**

In our single-center experience, the gap for competent trainee performance in essential early anesthetic management skills can be effectively covered by conducting an intensive, preparatory course using the combination of three educational strategies (e-learning, simulation-based hands on workshops, and observational learning) at the onset of residency. This course has allowed learning to be generated in a secure environment for both patients and trainees.

## Background

Ability to perform technical procedures in a proficient way is a major safety issue in anesthesia. Special concern [1] has emerged regarding the training process of new trainees before their first approach to patients in the anesthetic field. Worldwide, some institutions have established preparatory courses at the onset of residency for various specialties using simulation [2–5]; most of them only examine trainee self-reported preparedness instead of using task-specific tools for procedural skills assessments.

In Argentina, anesthesia training commences upon graduation from medical school; due to perceived deficits in procedural ability derived from undergraduate training, the researchers felt it was necessary to design and implement an intensive, preparatory course for our new anesthesiology trainees. As a first step towards integration of simulation-based learning into the anesthesiology training program, the decision was to sequentially combine three educational strategies and evaluate performance improvement in a stepwise approach on five procedural skills considered essential for early anesthetic management (peripheral intravenous cannulation, sterile hand wash and gowning, anesthesia workstation preparation, face-mask ventilation, and orotracheal intubation).

For acquisition of theoretical knowledge, e-learning has proven to be equivalent and possibly even superior than traditional learning, adding the advantage of self-paced completion of tasks [6]. Teaching procedural skills in scenarios allows the initial slope of the learning curve to take place while avoiding patient risk [7]. Simulation also extends an opportunity to get familiar with all segments of procedures and enables assessment of learners and corroboration of competence in scenarios before advancing onto patient practice. Vicarious or observational learning is defined as learning the appropriate visuo-motor behavior in a specific context by observing the actions of others and their outcomes [8].

Trainees have traditionally been assessed on procedural skills through retrospective feedback from supervising consultants without following specific criteria. International trends towards ensuring patient safety have inspired the development of tools for assessment of competence. The current best evidence supports assessment of procedural skills in anesthesia through direct observation by trained raters using task-specific tools; a combination of checklists and global rating scales (GRS) is suggested, especially when testing an intervention in educational research [9].

The primary aim of this study was to determine if a preparatory training course at the onset of anesthesiology residency is useful to achieve a competent trainee performance in the clinical setting. The secondary aims were to describe the trends in performance improvement between sequential assessment stages and compliance with predefined safety items for each procedural skill.

## Methods

Ethical approval for this prospective study was provided by the Ethical Committee of the Hospital Italiano de Buenos Aires, Buenos Aires, Argentina (Chairperson: Dr. Augusto Pérez, Ethical Committee N° 3389), on June 2017. The jurisdictional Institutional Review Board that approved the study established mandatory written consent from participants, which was obtained in all cases. The study subjects are new anesthesiology residents, who consented participation in staged assessments of competence and publication of obtained results avoiding individual identification. All recorded data was archived securely.

### Location and participants

The current investigation was a single-center, prospective, three-cohort study. It was conducted at a tertiary referral university-affiliated hospital in Buenos Aires, Argentina, from June 2017 to June 2019. Subjects included were anesthesia trainees from our program. Over three consecutive years, 24 residents comprising three cohorts of 8 residents each were involved in this course over a 4-week period at the onset of their residency; all trainees consented for participation in this research study, which derived in a total of 24 study participants. The course consisted of three training phases: an e-learning phase, a simulation-based hands on workshop phase, and an observational learning phase. These were combined with sequential assessments to evaluate whether and to what extent trainees achieved learning goals and how they progressed throughout the course.

### Training phases

The e-learning phase of the course was delivered through an online platform providing reading material, academic material, and audio-visual aids organized in modules for the selected skills. This phase lasted 14 days, during which trainees were granted deliberate access to the platform and were required to take multiple choice examinations for all modules. Obtaining passing qualifications on all modules allowed them to advance onto the workshop phase.

Before commencement of the workshop phase, participants went through baseline assessments (assessment stage 1) for the five selected procedural skills in the simulation center, which lasted 6–8 h. On the next day, the workshop phase in the simulation center began with a duration of 4 days. Simulation-based hands on workshops consisted of deliberate trainee practice with direct supervision and constant feedback. For peripheral intravenous cannulation, male multi-venous IV training arm kits (270-00001; Laerdal Medical Corporation, Wappingers Falls, NY, USA) were used. Cannulation techniques were demonstrated once and then 2 h deliberate practice with feedback followed. For sterile hand wash and gowning, trainees were handed operating room (OR) caps and surgical facemasks and were guided through hand wash, gowning, and gloving with deliberate practice and feedback. For anesthesia workstation preparation, the workshop was based on 2 h hands on practicing of skills for operating room preparation in a high-fidelity simulated OR. Face-mask ventilation and orotracheal intubation workshops were longer, divided in two parts. First, trainees were introduced on airway devices and accessories available at our institution. Secondly, 2 h deliberate practice of both procedures was allowed using airway management trainers (25000033; Laerdal Medical Corporation, Wappingers Falls, NY, USA). All materials were provided by the Anesthesiology Department, Hospital Italiano de Buenos Aires and CUESIM (Centro Universitario de Educación basado en Simulación, Instituto Universitario Hospital Italiano de Buenos Aires). As an additional activity, all trainees were put through one anesthetic scenario each, using the high-fidelity room equipped with a simulated OR and a simulation mannequin (212-02150 SimMan 3G; Laerdal Medical Corporation, Wappingers Falls, NY, USA). Eight individual scenarios were designed to integrate anesthesia workstation preparation, academic knowledge, and airway management skills. Assessments were not performed during these scenarios; mistakes and critical reflections were later addressed in 30 min debriefing group sessions. On the fifth day, all trainees went through assessment stage 2 in the simulation center.

The third phase of the course lasted 5 days and covered on-site vicarious observational learning in the OR setting. Trainees were assigned daily to an anesthesiology team (second to fifth year trainees accompanied by a consultant anesthesiologist) and held an observational role during the anesthetic activities of the day. On the sixth day, they went through the final assessment stage (assessment stage 3) during their first approach to patients in a supervised clinical setting (patients scheduled for elective surgery under general anesthesia and orotracheal intubation).

### Assessments

**Fig. 1:**
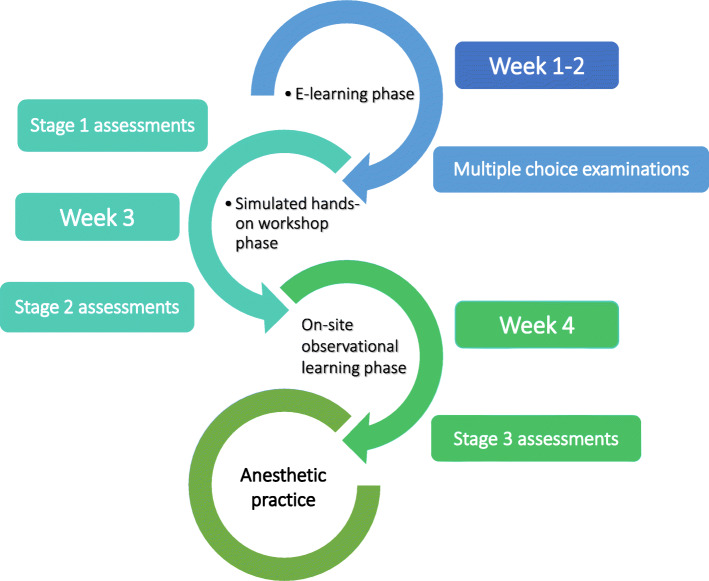
Flowchart

For the purpose of this investigation, participants went through individual, real-time assessments on a stepwise approach (three stages) applying task-specific checklists and a GRS for each procedure. Two physician anesthesiologists who were not involved as trainers in the course acted as raters. Both raters were given access to assessment tools in advance, and two simulated assessments were scheduled for raters training and calibration, before the course began. Trainees were randomized onto assessment by one of two raters; a comparable number of assessed trainees were reached by each rater. Selected procedures were peripheral intravenous cannulation, sterile hand wash and gowning, anesthesia workstation preparation, face-mask ventilation, and orotracheal intubation. Assessment stages 1 and 2 involved low and intermediate fidelity simulation models while stage 3 took place during trainees’ first supervised anesthetic practice on adult patients (Fig. 1).

The five procedural skills selected for assessment of competence were discussed by experts based on the frequency and autonomy on which these procedures are performed by new trainees. Assessments were performed through task-specific checklists and GRS on three occasions to assess individual competence for each skill; stage 1, to measure baseline performance on a simulated environment after the e-learning phase; stage 2, to measure performance on a simulated environment after addition of simulation-based hands on practice; and stage 3, to measure final performance in the clinical setting (on-site) as a result of transference of e-learning, simulation-based workshops, and observational learning to supervised patient care. For stage 3 assessments, appropriate patient candidates were identified, and trainees were scheduled accordingly. For sterile hand wash and gowning trainees were appointed to a senior resident or consultant anesthesiologist who was to perform an invasive sterile procedure and parallelly asked to demonstrate hand wash, gowning, and gloving and participate as a sterile observer through the procedure.

The two assessment tools (checklists and GRS) served complementary aims. Checklists emphasize on performing all steps of the procedure and in the correct sequence, while global rating scales focus on procedural behavior such as familiarity with procedure, efficiency of movements, or tissue care. All scores from checklists and GRS were used as percentages for statistical analysis and data presentation and were calculated using a 0/1 binary system in which total positive points were divided by the entire number of items.

Published task-specific validated checklists were used for assessment of peripheral intravenous cannulation [10], face-mask ventilation [11], and orotracheal intubation [12]. Specially designed checklists for anesthesia workstation preparation and sterile hand wash and gowning were developed according to our institutional practice guidelines (Additional files 2 and 3). Checklists presented either a binary (peripheral intravenous cannulation, sterile hand wash and gowning, and anesthesia workstation preparation) or a 3-point scoring system (face-mask ventilation and orotracheal intubation) for each item. For data analysis, item ratings were analyzed in a binary fashion. Hence, ratings of 1 or 2 on face-mask ventilation checklist by Ahmed et al. and “no” or “yes but” on orotracheal intubation checklist by Walzak et al. were considered as not correctly performed and given 0 points in our database, whereas ratings of 3 and “yes,” respectively, were given 1 point. For orotracheal intubation during all assessment stages, number 4 Macintosh blades from C-MAC videolaryngoscope were used. Therefore, trainees performed direct laryngoscopy and raters could indirectly visualize the procedure through the C-MAC separate video monitor screen which, in addition to capnography tracing, allowed for confirmation of intubation success.

A variable number of safety items were defined for each checklist by expert consensus. Compliance with these safety items was quantified for each procedural skill assessed in stage 3 as a secondary aim of the study.

A global rating scale (GRS), first published by Martin et al. in 1997 [13] is based on the Objective Structured Assessment of Technical Skills (OSATS) [14] tool used to evaluate surgical residents’ technical skills [15]. It has also been extensively validated for evaluation of anesthetic skills [16–18]. It is a nine-item, 5-point text-anchored Likert scale form which permits evaluators to appraise multiple approaches of skill performance. This tool was applied individually to the five selected tasks during all assessment stages. For two of the procedures (sterile hand wash and gowning and anesthesia workstation preparation), the patient care item was excluded.

The primary outcome measure was achievement of competent scores on task-specific checklists and GRS during final assessments. Competent scores were defined as scores higher than 85% derived from results of two simulated assessments involving post-graduate year 5 (PGY-5) anesthesia trainees; these simulated assessments were designed for rater training and definition of competence threshold [19]. Secondary outcomes were performance improvement between scores from assessment stages 1–2 and stages 2–3, which were analyzed along with overall performance improvement (between stages 1 and 3) and compliance with safety items during stage 3.

### Statistical analysis

For each of the five assessed procedural skills, final scores (assessment stage 3) were evaluated for achievement of competence. In addition, three differences between sequential scores were calculated (performance improvement) through *t* test for paired data: between baseline scores (stage 1) and after workshop scores (stage 2), between after workshop scores (stage 2) and final scores (stage 3), and overall performance improvement (between stages 1 and 3). As three measurements were analyzed through *t* test for paired data, statistical significance was defined as *p* value < 0.016.

## Results

The study participants included a total of 24 anesthesia trainees who enrolled and completed the study. The entire trainee cohort participated every year, with a male/female proportion of 15/9; none of the participants admitted having previous anesthetic training. In Argentina, medical school does not systematically provide training for any of the procedural skills approached in this study. Twenty trainees (83.3%) were found to be globally competent (both assessment tools for all procedures) during final assessments (stage 3).

On checklist assessments stage 3, 24 trainees (100%) achieved competent scores on peripheral intravenous cannulation, 23 (96%) on sterile hand wash and gowning [trainee number 3 failed to achieve competence], 23 (96%) on anesthesia workstation preparation [trainee number 14 failed to achieve competence], 24 (100%) on face-mask ventilation, and 23 (96%) on orotracheal intubation [trainee number 19 failed to achieve competence]. Three different trainees (trainee numbers 3, 14, and 19) failed to achieve competence in only one procedure (sterile hand wash and gowning, anesthesia workstation preparation, and orotracheal intubation, respectively).

For GRS assessments stage 3, 21 trainees (87.5%) achieved competent scores on peripheral intravenous cannulation [trainee numbers 3, 9, and 14 failed to achieve competence], 21 (87.5%) on sterile hand wash and gowning [trainee numbers 3, 14, and 19 failed to achieve competence], 22 (92%) on anesthesia workstation preparation [trainee numbers 14 and 19 failed to achieve competence], 20 (83.3%) on face-mask ventilation [trainee numbers 3, 9, 14, and 19 failed to achieve competence], and 20 (83.3%) on orotracheal intubation [trainee numbers 3, 9, 14, and 19 failed to achieve competence]. Four trainees (trainee numbers 3, 9, 14, and 19) failed to achieve competence in more than one procedure (Table 1).

**Table 1:** Achievement of competence for checklists and GRS assessments for five procedural skills

Procedural skill	Competence achievement: checklist assessments (*n* = 24)	Competence achievement: GRS assessments (*n* = 24)
Peripheral intravenous cannulation	24 (100%)	21 (87.5%)
Sterile hand wash and gowning	23 (96%)	21 (87.5%)
Anesthesia workstation preparation	23 (96%)	22 (92%)
Face-mask ventilation	24 (100%)	20 (83.3%)
Orotracheal intubation	23 (96%)	20 (83.3%)

Mean baseline (stage 1), after workshop (stage 2), and final (stage 3) scores from 24 participants presented as percentages are available in Table 2. Overall performance improvement (between stages 1 and 3) was found to be statistically significant for all procedural skills assessed by checklists and GRS (*p* < 0.001). During stage 3, 21 trainees (87.5%) complied with all safety items defined on checklists for the five procedural skills assessed.

**Table 2:** Checklist and GRS staged scores for procedural skills assessed.

Procedural skill	Checklist scores			Global rating scale scores		
	Stage 1	Stage 2	Stage 3	Stage 1	Stage 2	Stage 3
Peripheral intravenous cannulation	62.2 ± 10.3	71.7 ± 7.8	93.9 ± 3.9	56.1 ± 10.4	77.4 ± 13.4	89.4 ± 9.7
Sterile hand wash and gowning	63.9 ± 26.3	90.3 ± 13.8	97.2 ± 13.6	55.7 ± 13.3	83.8 ± 14.1	90.95 ± 9.3
Anesthesia workstation preparation	48.5 ± 22.7	84.8 ± 13.3	96.2 ± 8	44.2 ± 21.7	73.8 ± 16.3	93.8 ± 7.66
Face-mask ventilation	55.5 ± 24.4	73.6 ± 20.2	100	49.7 ± 14.3	79.8 ± 15.5	87.9 ± 11.1
Orotracheal intubation	61.4 ± 15.7	65 ± 8.2	90.8 ± 7.8	49.6 ± 14.5	77.3 ± 14.4	88.5 ± 10

Data are expressed as the mean ± standard deviation

## Performance improvement between assessment stages 1 and 2

For checklist assessments, performance improvement between stages 1 and 2 was statistically significant for peripheral intravenous cannulation, sterile hand wash and gowning, anesthesia workstation preparation, and face-mask ventilation. Performance improvement was not statistically significant for orotracheal intubation. For GRS assessments, performance improvement between stages 1 and 2 was statistically significant for all procedural skills (Table 3).

**Table 3:** Performance improvement between stages 1 and 2 for checklists and GRS assessments

Procedural skill	Performance improvement between stages 1 and 2	
	Checklist assessments	GRS assessments
Peripheral intravenous cannulation	9.5 [6.1 to 13]*	21.25 [16.3 to 26.2]*
Sterile hand wash and gowning	26.4 [16.9 to 35.9]*	28.1 [22.6 to 33.6]*
Anesthesia workstation preparation	36.4 [27 to 45.7]*	29.6 [22.4 to 36.9]*
Face-mask ventilation	18.1 [10.72 to 25.4]*	30.3 [25 to 35.6]*
Orotracheal intubation	3.6 [− 2.25 to 9.5]ˠ	27.7 [21.2 to 34.2]*

Results are presented as mean difference [CI 95%]

*p < 0.001

ˠ*p* = 0.203

## Performance improvement between stages 2 and 3

For checklist assessments, performance improvement between stages 2 and 3 was found to be statistically significant for peripheral intravenous cannulation, anesthesia workstation preparation, face-mask ventilation, and orotracheal intubation. Performance improvement was not statistically significant for sterile hand wash and gowning. For GRS assessments, performance improvement between stages 2 and 3 was found to be statistically significant for all procedural skills (Table 4).

**Table 4:** Performance improvement between stages 2 and 3 for checklists and GRS assessments

Procedural skill	Performance improvement between stages 2 and 3	
	Checklist assessments	GRS assessments
Peripheral intravenous cannulation	22.2 [19.3 to 25]*	12 [7.6 to 16.4]*
Sterile hand wash and gowning	6.9 [0.7 to 13.1]^x^	7.1 [2.8 to 11.4]*
Anesthesia workstation preparation	11.4 [5.8 to 16.9]*	20 [14.9 to 25.1]*
Face-mask ventilation	26.4 [18.3 to 34.5]*	7.9 [2.5 to 13.3]□
Orotracheal intubation	25.8 [21.4 to 30.2]*	11.25 [5.9 to 16.5]*

Results are presented as mean difference [CI 95%]

*p < 0.001

^x^*p* = 0.029

□*p* = 0.006

## Discussion

This preparatory course was designed as our first step towards integration of simulation-based learning into the anesthesiology training program. Results from the present analyses largely support the idea that trainee performance, and hence patient safety, can be improved through introductory combined educational strategies and simulation-based practice ahead of patient care.

Most trainees were found to achieve competent scores when performing procedural skills on patients (assessment stage 3). Statistically and clinically significant differences were found when evaluating performance improvement between sequential assessments for four procedures between stages 1 and 2 (except for checklist assessment of orotracheal intubation) and for four procedures between stages 2 and 3 (except for checklist assessment of sterile hand wash and gowning), corroborating the need for diverse, consecutive phases of training and assessment in order to achieve trainee competent performance. Therefore, one of the goals of this course, transference of skills from e-learning, simulation-based workshops, and vicarious observational learning onto anesthesiology practice, was accomplished.

Non-statistically significant performance improvement was found between stages 1 and 2 for orotracheal intubation. Mean difference between these stages was 3.6 (95% CI − 2.25 to 9.5; *p* = 0.215). This result does not entirely surprise the authors since it is a complex procedural skill which requires a combination of cognitive and psychomotor components probably not present at this point in training. Nonetheless, statistically significant performance improvement between stages 2 and 3 and achievement of competence during final assessments suggest that the addition of vicarious observational learning boosts performance for this skill. Moreover, 23 trainees (96%) complied with all safety items in the final assessment stage for this skill.

Focusing on trends of performance improvement between stages, the results indicate that for skills involving patient care (like peripheral intravenous cannulation and airway skills), improvement is higher through observational on-site learning when assessed by checklists. For patient care skills, observational learning seems to be essential for integration of knowledge and practice from the course with maneuvers imitated from more experienced physicians. The authors assume this may be related to intermediate fidelity provided by cannulation arms and airway trainers. On the contrary, for those skills which do not involve patient care (like anesthesia workstation preparation and sterile hand wash and gowning), improvement is higher through hands on workshops. In these cases, the authors believe that high fidelity of simulated OR environments designed for these workshops was key to achieve high scores on stage 2 assessments, leaving observational learning a small place for improvement as in sterile hand wash and gowning [19].

For all skills assessed by GRS, performance improvement was higher between stages 1 and 2 than between stages 2 and 3. The GRS by Martin et al. is based on items like time, motion, and instrument handling, among others; it was expected by authors that these aptitudes would improve after simulation-based hands on deliberate practice. Nonetheless, improvement between stages 2 and 3 was also statistically significant for all procedures. In consequence, the authors can state that both strategies are useful to learn context-dependent aspects of skills.

Our results suggest that vicarious observational learning can have substantial impact on procedural skills training. As explained by Nehls et al. [20], for vicarious observational learning to take place, situational engagement is required, which the authors believe was accomplished by daily scheduling trainees together with a superior trainee and a consultant anesthesiologist on site, in the OR. During this 5-day phase, each trainee held a mere observational role in approximately 15–20 cases of general anesthesia (data obtained from surgical timetable) [21]. For orotracheal intubation, we can imply from the present study that watching the task being performed by a non-expert supervised operator probably allows them to map other’s actions onto their own, enhance performance, and avoid unnecessary errors [8]. Consequently, for healthcare procedural skills training, vicarious observational learning should always immediately precede direct experiential learning.

Many hospitals throughout the world might expect trainees to have some experience with hand washing, gowning, and peripheral intravenous cannulation, which could be considered as generic skills. These are not addressed during undergraduate training in our medical system and for this reason their inclusion in our list of essential skills.

The major limitation of the present work is the lack of comparison with a control group. In the need for safer training methods for anesthesiology trainees ahead of patient care, the Anesthesiology Department decided to include all trainees in this preparatory course and simultaneously evaluate the learning outcomes, leaving no place for a control group. Another limitation of our work may be the relatively small number of participants. Trainees were randomized to undergo evaluation by one of the two trained raters. Assessment outcomes by different raters were not analyzed for interrater consistency; this decision was sustained by high interrater reliability coefficients reported in the literature for these tools (0.99, 0.95, and 0.88 for peripheral venous cannulation, face-mask ventilation, and orotracheal intubation, respectively). Checklists for anesthesia workstation preparation and sterile hand wash and gowning were not tested for interrater reliability. Finally, this is a single-center study; it is not feasible to generalize our findings to all anesthesiology training programs. Despite that, it is possible that all kinds of learners may benefit from similar courses or preparatory sessions ahead of patient care.

Other published reports have already aimed for a safer healthcare environment through pre or early residency training programs like boot camps or intensive introductory courses. Nonetheless, the uniqueness of this program relies on a prospectively designed assessment methodology to determine if, and when, trainees were able to demonstrate competence on the skills that the program intended to teach. This process should probably be linked to any program modifications or extensions. Additionally, a combination of consecutive learning strategies has not been reported in the literature. Sequential assessments using a combination of tools make it possible to calculate and analyze which strategies serve as the most effective training method for the five selected skills. Finally, unlike other studies, a distinctive contribution of this investigation is that final assessments were performed during trainees’ first approach to patients, which permitted evaluation of transference of skills acquired throughout the course onto clinical practice.

This work has potentially high-impact implications in anesthesiology residency programs, showing that trainees can competently perform skills in clinical practice that they have learned through simulation, which is still relatively uncommon nowadays.

## Conclusion

In conclusion, the need for an intermediate training phase between medical school graduation and beginning of a post-graduate program in anesthesiology is presented here; this study demonstrates that in our single-center experience, the gap for essential early anesthetic management skills can be effectively covered by conducting an intensive, preparatory course using the combination of three educational strategies (e-learning, simulation-based hands on workshops, and observational learning) at the onset of residency. In addition, our institution offers high-quality patient care with special focus on safety. This course has allowed learning to be generated in a secure environment for both patients and trainees.

## Supplementary information

**Additional file 1:** Chronological program. Chronologically organized program including detailed material for the e-learning phase modules (available reading, academic and audio-visual material for trainees during this phase).

**Additional file 2:** Anesthesia workstation preparation checklist. Designed checklist for assessment of anesthesia workstation preparation skills based on Institutional Guidelines (Hospital Italiano de Buenos Aires). Yes/no binary scoring system.

**Additional file 3:** Sterile hand wash and gowning checklist. Designed checklist for assessment of sterile hand wash and gowning skills based on Institutional Guidelines (Hospital Italiano de Buenos Aires). Yes/no binary scoring system.

## Data Availability

The datasets used and/or analyzed during the current study are available from the corresponding author on reasonable request.

## Abbreviations

GRS: Global rating scale
OR: Operating room
CUESIM: Centro Universitario de Educación basado en Simulación
CI: Confidence interval
PGY-5: Post-graduate year 5
