# Supplementary material for: Simulation-based training for early procedural skills acquisition in new anesthesia trainees: a prospective observational study
Source: Adv Simul (Lond). 2020 Aug 12;5:19. doi: 10.1186/s41077-020-00135-z (PMC7424643; doi:10.1186/s41077-020-00135-z)
Supplement: Supplementary file 1 — Additional file 1: Chronological program. Chronologically organized program including detailed material for the e-learning phase modules (available reading, academic and audio-visual material for trainees during this phase). [file 41077_2020_135_MOESM1_ESM.docx]

***Additional file 1***

1. *WEEK 1-2: E-LEARNING PHASE.*

The e-learning phase of the course was delivered through an online platform providing reading material, academic material and audio-visual aids organized in modules for the selected skills. This phase lasted fourteen days, during which trainees were granted deliberate access to the platform and were required to take multiple choice examinations for all modules. Obtaining passing qualifications on all modules allowed them to advance onto the workshop phase.

1.1. **Preoperative assessment**. Reading and academic material focused on guidelines for preoperative assessment from the Anesthesiology Department and guideline by ESA (European Society of Anaesthesiology): Preoperative evaluation of the adult patient undergoing non-cardiac surgery: guidelines from the European Society of Anaesthesiology.

1.2. **Anesthesia workstation preparation**. Institutional practice guidelines on anesthesia workstation preparation and OR equipment (Additional file 2).

1.3. **Basic Anesthetic Monitoring**. Reading material: Standards for Basic Anesthetic Monitoring developed by the Committee on Standards and Practice Parameters (CSPP), ASA (American Society of Anesthesiology) and Recommendations for standards of monitoring during anaesthesia and recovery 2015, by the Association of Anaesthetists of Great Britain and Ireland.

1.4. **Peripheral venous and arterial cannulation.** Reading material from New England Journal of Medicine, *VIDEOS IN CLINICAL MEDICINE* (1-4) and institutional video guidance trainees on peripheral intravenous cannulation.

*1.4.1. Peripheral Intravenous Cannulation* (Rafael Ortega, M.D., Pavan Sekhar, M.D., Michael Song, M.D., Christopher J. Hansen, B.A., and Lauren Peterson; From the Department of Anesthesiology, Boston Medical Center, Boston. N Engl J Med 2008;359:e26)

*1.4.2. Arterial Puncture for Blood Gas Analysis* (Shelly P. Dev, M.D., Melinda D. Hillmer, M.D., BSc.Phm., and Mauricio Ferri, M.D.; From the Department of Critical Care Medicine, Sunnybrook Health Sciences Centre, University of Toronto, Toronto. N Engl J Med 2011;364:e7).

*1.4.3. Central Venous Catheterization* (Alan S. Graham, M.D., Caroline Ozment, M.D., Ken Tegtmeyer, M.D., Susanna Lai, M.P.H., and Dana A.V. Braner, M.D. From the Department of Pediatrics, Division of Pediatric Critical Care, Doernbecher Children’s Hospital, Portland, OR (A.S.G., K.T., S.L., D.A.V.B.); and the Department of Pediatrics, Division of Pediatric Critical Care, Duke University, Durham, NC (C.O.). N Engl J Med 2007;356:e21).

*1.4.4. Ultrasound-Guided Internal Jugular Vein Cannulation* (Rafael Ortega, M.D., Michael Song, M.D., Christopher J. Hansen, M.A., and Paul Barash, M.D.; From the Department of Anesthesiology, Boston Medical Center, Boston (R.O., M.S., C.J.H.); and the Department of Anesthesiology, Yale School of Medicine, New Haven, CT (P.B.). N Engl J Med 2010;362:e57.

1.5. **Airway management.** Reading material: Institutional guidance for trainees on basic and advanced airway management by Dr. Gonzalo Domenech including: airway preoperative assessment, face-mask ventilation, laryngoscopy and oro/nasotracheal intubation procedures, awake intubation, emergency invasive airway access).

1.6. **Preparation and management of basic anesthetic drugs.** Reading material by Dr. Hernan Boveri. Video focused on setting of syringe pumps.

1.7. **Electronic anesthetic record.** Reading material focused on institutional electronic medical record and Anesthetic records.

1.8. **Sterile hand wash and gowning**. Reading material focused on institutional practice guidelines for *The Joint Commission International for Anesthesiology and Institutional subsections*, [Antibiotic Prophylaxis to Prevent Surgical Site Infections](https://www.aafp.org/afp/2011/0301/p585.html#:~:text=Prophylactic%20antibiotic%20administration%20should%20be,is%20receiving%20vancomycin%20or%20fluoroquinolones.&text=Antibiotic%20prophylaxis%20should%20be%20appropriate,and%20consistent%20with%20SCIP%20guidelines.) and catheter infections, hand hygiene, sterile hand wash, gowning and maintenance of sterility during procedures.

1.9. Multiple choice examinations for all modules. Obtaining passing qualifications on all modules allowed them to advance onto the workshop phase.

1. *WEEK 3: WORKSHOP PHASE*

*2.1.* ***Stage 1 assessments (8 hours)***

*2.2.* **Simulated hands on workshops (4 days)**

*2.2.1.* Peripheral intravenous cannulation: using male multi-venous IV training arm kits ([270-00001](http://www.laerdal.com/item/270-00001); Laerdal Medical Corporation, Wappingers Falls, NY, US) were used. Cannulation techniques were demonstrated once and then two-hour deliberate practice with feedback by the workshop instructor followed.

*2.2.2.* Sterile hand wash and gowning: trainees were handed operating room (OR) caps and surgical facemasks and were guided through hand wash, gowning, and gloving with deliberate practice and feedback by the workshop instructor.

*2.2.3.* Anesthesia workstation preparation: the workshop was based on two-hour hands-on practicing of skills for operating room preparation in a high-fidelity simulated OR and feedback by the workshop instructor.

*2.2.4.* Face-mask ventilation and orotracheal intubation workshops. First, trainees were introduced on airway devices and accessories available at our institution. Secondly, two-hour deliberate practice of both procedures was allowed using airway management trainers (25000033; Laerdal Medical Corporation, Wappingers Falls, NY, US) for face-mask ventilation and orotracheal intubation by direct and videolaryngoscopy followed by feedback by the workshop instructor.

2.3. ***Stage 2 assessments (8 hours)***

1. *WEEK 4: OBSERVATIONAL LEARNING PHASE*

On-site vicarious observational learning in the OR setting during 5 days. Trainees were assigned daily to an anesthesiology team (second to fifth-year trainees accompanied by a consultant anesthesiologist) and held an observational role during the anesthetic activities of the day.

3.1. ***Stage 3 assessments (8 hours)***
