# Supplementary material for: Simulation-based training for early procedural skills acquisition in new anesthesia trainees: a prospective observational study
Source: Adv Simul (Lond). 2020 Aug 12;5:19. doi: 10.1186/s41077-020-00135-z (PMC7424643; doi:10.1186/s41077-020-00135-z)
Supplement: Supplementary file 2 — Additional file 2: Anesthesia workstation preparation checklist. Designed checklist for assessment of anesthesia workstation preparation skills based on Institutional Guidelines (Hospital Italiano de Buenos Aires). Yes/no binary scoring system. [file 41077_2020_135_MOESM2_ESM.docx]

- **Anesthesia workstation preparation based on Institutional Guidelines (Hospital Italiano de Buenos Aires)**

|  | Y | N |
| --- | --- | --- |
| 1. Check gas outlets and oxygen supply (distinguish pipes between O2, air and other gases) * |  |  |
| 1. Check emergency oxygen supply and rotameter |  |  |
| 1. Check airway mask-bag unit (AMBU) availability |  |  |
| 1. Anesthesia machine turned on and software check confirmed |  |  |
| 1. Gas leak test performed* |  |  |
| 1. Functioning and prepared suction* |  |  |
| 1. Check airway equipment availability (face mask, oropharyngeal cannula, functioning laryngoscope with appropriate blades, endotracheal tubes, stethoscope) |  |  |
| 1. Check difficult airway kit availability* |  |  |
| 1. Check standard monitoring availability (pulse oximetry, non-invasive pressure cuff, 5-lead electrocardiogram, gas analyser) |  |  |
| 1. Check basic anesthetic induction drugs availability (hypnotic, neuromuscular blockade drug, analgesic, maintenance drug, infusion pumps, emergency vasoactive drugs) |  |  |
| 1. Check defibrillator availability and ACLS drugs |  |  |

**Safety items*

*Reference:*

- Connor D, Collis R, Coley E, Greatorex B, Hodges S, James J, McGuire N, Mercer S, Neighbour R, Sheraton T, Walker I. Checklist for draw-over anaesthetic equipment 2019. In: [Resources & publications](https://anaesthetists.org/Home/Resources-publications), [Guidelines](https://anaesthetists.org/Home/Resources-publications/Guidelines). Association of Anaesthetists of Great Britain & Ireland. 2019. <https://anaesthetists.org/Home/Resources-publications/Guidelines/Checklist-for-draw-over-anaesthetic-equipment-2019>. Accessed 19 June 2020.
