# Supplementary material for: Simulation-based training for early procedural skills acquisition in new anesthesia trainees: a prospective observational study
Source: Adv Simul (Lond). 2020 Aug 12;5:19. doi: 10.1186/s41077-020-00135-z (PMC7424643; doi:10.1186/s41077-020-00135-z)
Supplement: Supplementary file 3 — Additional file 3: Sterile hand wash and gowning checklist. Designed checklist for assessment of sterile hand wash and gowning skills based on Institutional Guidelines (Hospital Italiano de Buenos Aires). Yes/no binary scoring system. [file 41077_2020_135_MOESM3_ESM.docx]

- **Sterile hand wash and gowning based on Institutional Guidelines**

|  | Y | N |
| --- | --- | --- |
| - - - 1. Correctly don OR hair cover |  |  |
| - - - 1. Correctly don surgical facemask |  |  |
| - - - 1. Correct technique for sterile handwash |  |  |
| - - - 1. Correct technique for sterile gowning |  |  |
| - - - 1. Correct technique for sterile gloving |  |  |
| - - - 1. Always maintains sterile * |  |  |

**Safety items*

*Reference:*

- Soule BM. Evidence-Based Principles and Practices for Preventing Surgical Site Infections. Joint Commission International, Oakbrook Terrace, IL; 2018.
